# Robot-Aided Mapping of Wrist Proprioceptive Acuity across a 3D Workspace

**DOI:** 10.1371/journal.pone.0161155

**Published:** 2016-08-18

**Authors:** Francesca Marini, Valentina Squeri, Pietro Morasso, Jürgen Konczak, Lorenzo Masia

**Affiliations:** 1 Motor Learning and Robotic Rehabilitation Laboratory, Department of Robotics, Brain and Cognitive Sciences, Istituto Italiano di Tecnologia, Genova, Italy; 2 Human Sensorimotor Control Laboratory, School of Kinesiology and Center for Clinical Movement Science, University of Minnesota, Minneapolis, Minnesota, United States of America; 3 School of Mechanical & Aerospace Engineering, Nanyang Technological University, Singapore, Singapore; Tianjin University, CHINA

## Abstract

Proprioceptive signals from peripheral mechanoreceptors form the basis for bodily perception and are known to be essential for motor control. However we still have an incomplete understanding of how proprioception differs between joints, whether it differs among the various degrees-of-freedom (DoFs) within a particular joint, and how such differences affect motor control and learning. We here introduce a robot-aided method to objectively measure proprioceptive function: specifically, we systematically mapped wrist proprioceptive acuity across the three DoFs of the wrist/hand complex with the aim to characterize the wrist position sense. Thirty healthy young adults performed an ipsilateral active joint position matching task with their dominant wrist using a haptic robotic exoskeleton. Our results indicate that the active wrist position sense acuity is anisotropic across the joint, with the abduction/adduction DoF having the highest acuity (the error of acuity for flexion/extension is 4.64 ± 0.24°; abduction/adduction: 3.68 ± 0.32°; supination/pronation: 5.15 ± 0.37°) and they also revealed that proprioceptive acuity decreases for smaller joint displacements. We believe this knowledge is imperative in a clinical scenario when assessing proprioceptive deficits and for understanding how such sensory deficits relate to observable motor impairments.

## Introduction

Proprioceptive signals originate from mechanoreceptors within muscles, tendons, and skin, which give rise to kinaesthesia (the sense of limb movement) and the sense of joint position. These afferent signals are not only crucial for bodily awareness, but they have great impact on voluntary motor control, and on the regulation of muscle tone and postural stability [1–4]. Numerous neurological diseases, such as cortical stroke or Parkinson’s disease [5, 6], are known to impair proprioception which negatively affects posture, limbs movement control and motor learning [7, 8], therefore, the assessment and quantification of proprioceptive function has long been recognised as important for diagnosis and for determining therapeutic efficacy. Yet, at present, there is no established objective method for its clinical assessment, and despite clinical rating scales such as the Nottingham Sensory Assessment [9] or the Rivermead Assessment of Somatosensory Performance [10] are available and currently in use, they can provide only qualitative information and have low resolution. Other tests have been developed in recent years, and among them, two specifically have been gaining interest: the *Joint Position Matching* (JPM) test [11], and the *Psychophysical Threshold Method* (PTM) [12]. The JPM measures the accuracy in replicating a joint angle in absence of vision [13], while the PTM quantifies subject’s sensitivity in discriminating the largest amplitude between two passive movements [14].

Current limitation for the application of these tests is the scarce presence of objective measurement technology in many clinical setting. The commonly used tools, such as hand-held goniometers, are known to lack of sensitivity and reliability. Recent advancements in haptic interfaces designed for sensorimotor rehabilitation provided the starting point for an innovative robot-aided approach for the assessment of proprioceptive functions [15]. The possibility to implement the JPM and PTM tests on robotic platforms allows for collection of large normative data sets through a reliable procedure that yields objective, reliable data at a high resolution. Previous contributions highlighted the efficacy of robotic devices in providing meaningful information on proprioceptive sensitivity for both healthy [16] and neurological subjects [17, 18], but most of them focused on the proximal upper limb joints (shoulder and elbow [19, 20] or elbow alone [21]) and lower limb (ankle and knee [22]).

One of the few evidences on robot-aided assessment of wrist proprioception used a single degree of freedom (DoF) PTM test for the flexion/extension (FE) [23], while no data were provided for the two remaining wrist DoFs (abduction/adduction (AA) and pronation/supination(PS).

Moreover, the suitability of passive tests for evaluation of joint position sense has been previously challenged [24], contending that the proprioceptive system only functions properly when muscles contraction occurs for voluntary action or stretch reflexes [25]. Previous contributions suggested that active test (like the JPM) results may have more relevance than passive ones because most of daily functions involves voluntary or reflex muscle control [26]; furthermore a passive test like the PTM may not result suitable in clinical practice because of the large amount of time needed to gather reliable measures, leading to a decrement in subjects’ attention which can modify afferent signals [27] and introduce a time dependent quality of the results. Therefore, in order to provide a complete and quantitative assessment of wrist proprioception, with an accurate and reliable test, quick and easy to administer, we implemented on a robotic wrist device the JPM test. Thirty healthy subjects were enrolled in the study that had a twofold aim: quantify and compare the proprioceptive acuity of all the three wrist degrees of freedom (FE, AA and PS), and examine different angular configurations to understand how proprioceptive acuity may change dependently on the amplitude of the wrist angles to perceive [28, 29].

Results indicated a consistent anisotropy of proprioceptive acuity across the three DoFs, with the AA being more accurate and precise than FE and PS. Additionally, we found that proprioceptive acuity changes dependent on movement amplitudes and sensitivity is higher at the limit of the functional wrist Range of Motion (RoM).

## Materials and Methods

### Participants and robotic device

Thirty right-handed subjects (mean age 28.9 ± 3.9 years, 16 females, 14 males), with no history of neuromuscular disorders and naïve to the task, participated to the study. Handedness of all participants was assessed using the Edinburgh Handedness Inventory [30] and the study was approved by ethics committee of the regional health authority, Azienda Sanitaria Locale Genovese (ASL) N.3 (Protocol number 29/08 approved on 10/2/2008). Experiments were carried out at the Motor Learning and Robotic Rehabilitation Lab of the Istituto Italiano di Tecnologia (Genoa, Italy). Each subject signed a consent form that conforms to these guidelines, according to ethics committee requirements.

### Procedure

**Fig 1:**
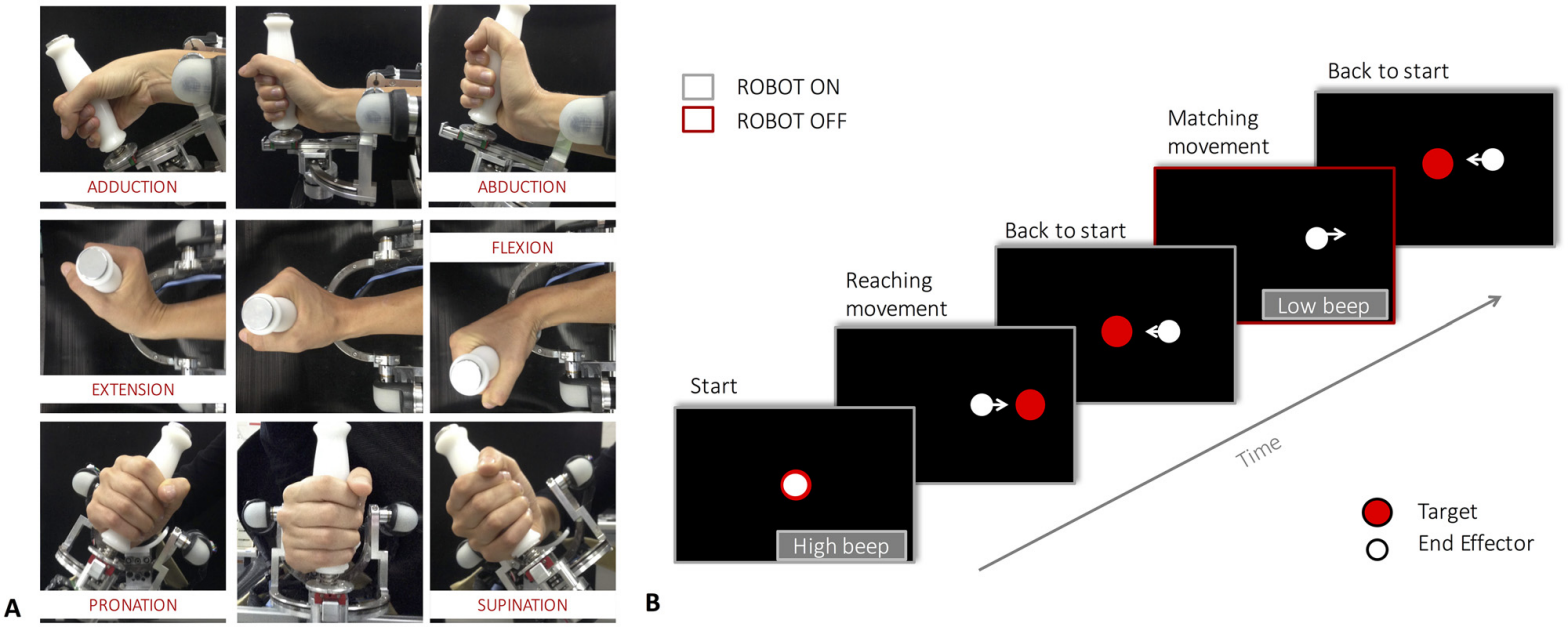
A) Wrist’s DoFs and movements involved in the task (flexion/extension, abduction/adduction and pronation/supination), with reference to an initial neutral position. Wrist motion is measured/actuated by the robotic exoskeleton shown in the figure. B) The temporal sequence of the experimental paradigm. An auditory cue marks the beginning of the trial and the wrist is passively moved by the robotic device from the neutral configuration to the proprioceptive target. After a consistent holding time of 3 seconds, the joint is passively returned to the initial starting position. Another auditory cue indicates participants to start moving and actively reproduce, the joint configuration previously experienced. In this phase the robot is inactive. When the end effector speed is below a 2°/second for more than 2 seconds, the robot moves the wrist back to the neutral position and another trial can start.

Participants sat in front of a three DoFs wrist manipulandum [31] holding its handle with their right hand; the robotic device allowed for movements along the three DoFs of the wrist (Fig 1A) at nearly the full range of motion of the respective joints. The robot was powered by four brushless motors that provided high transparency, accurate haptic rendering and compensate for the weight and inertia of the device during movements, while angular rotations on the three axes were measured by means of high resolution incremental encoders. Subjects’ frontal plane was perpendicularly aligned to the PS axis of the robotic device and arm position was adjusted to have a 90° angle between the upper and the forearm. Particular attention was given to the correct alignment between the axes of the robotic system and the anatomical axes of the wrist/forearm complex: the subjects’ forearm was firmly strapped to a mechanical support to ensure repeatability of wrist positioning across the different trials and to avoid joints misalignment or unwanted relative movement during task execution. Proprioceptive acuity was assessed with an ipsilateral joint position matching (JPM) procedure (Fig 1B). Starting from the neutral anatomical position (0° of FE, 0° of AA and 0° of PS), a reference position consisting in a preset angular displacement (or proprioceptive target) was initially presented to the participant by passively moving his/her wrist by the robot [32, 33] to the reference position and then holding the position for three seconds. Subsequently, the joint was moved back to the neutral position, and the participant was requested to actively move the wrist to the previously experienced position with no assistance from the device.

Participants did not receive any performance feedback during testing. Vision was occluded with opaque goggles to prevent online visual feedback of the wrist position.

Each of the three wrist’s DoFs was tested separately: participants had to match proprioceptive targets located along each of them, at a distance chosen as 80% of the total functional wrist’s RoM (indicated as large workspace, LWS). In particular, these position were: 32° (80% of 40°) for Flexion and Extension; 16° (80% of 20°) for Abduction and Adduction; 24° (80% of 30°) for Pronation and Supination. Proprioceptive targets were presented 12 times for each DoF for a total of 36 trials in a pseudorandom fashion. During each trial the robot allowed movements only in the tested DoF, while holding the other two DoFs in the neutral configuration (i.e. when FE is passively moved the PS and AA are blocked).

In order to determine if proprioceptive acuity depends on amplitude of the experienced target, wrist position sense was also tested in a smaller workspace (SWS). Proprioceptive targets in the small workspace were located at 40% of the functional RoM (50% of those in the LWS): 16° for Flexion and Extension, 8° for Abduction and Adduction, and 12° for Pronation and Supination. Also for the SWS condition participants experienced 12 target for each of the 3 directions for a total of 36 trials. Overall, the test consisted in 72 trials, with a total duration of 45 minutes.

### Outcome measures

Wrist joint rotations were recorded from the robot’s incremental encoders; acquired signals were post-processed by a third-order Savitzky-Golay low-pass filter (cut-off frequency of 10 Hz) and converted into angular displacements from the direct kinematic of the robot. To estimate the proprioceptive acuity of the wrist position sense and characterise the overall performance, actual (active) and desired (passive) wrist positions were compared. In order to capture the breadth of errors observed during careful inspection of the data from many participants, we calculated 3 measures that reflected distinctive patterns of errors commonly observed: the *matching error* (ME) [34] to quantify the overall accuracy, the *error bias* (EB) [34, 35] that provides the average error in responding considering its direction, and the *variability* (V) [34, 35] to quantify matching consistency/inconsistency across the trials.

The *matching error* is computed by averaging over the N(= 12) trials repeated under identical condition (same DoF and workspace), the absolute value of the angular deviation from the proprioceptive target:

$$\begin{array}{c} \text{ME}=\frac{\sum_{i=1:N}\left|\theta_{i}-\theta_{T}\right|}{N} \end{array}$$ (1)

where *θ*_*i*_ is wrist’s final position of the *i*-trial, *θ*_*T*_ is the proprioceptive target position and N is the number of trials in the tested DoF.

The *Variability*, *V* is evaluated, for each DoF, as the standard deviation across the 12 trials of wrist’s position at the end of the matching movement and it provides information about performance consistency (precision) across the whole experiment:

$$\begin{array}{c} V=StD(\theta_{i=1:N}) \end{array}$$ (2)

The *error bias* provides information about a performer’s response bias: it is the directional distance evaluated as algebraic summation between the ideal proprioceptive target and the actual wrist position, indicating the subjects’ tendency in undershooting (negative error bias) or overshooting (positive error bias) the target.

$$\begin{array}{c} \text{EB}=\frac{\sum_{i=1:N}\left(\theta_{i}-\theta_{T}\right)}{N} \end{array}$$ (3)

The *matching error* indicates error amplitude and is a direct measure of proprioceptive acuity. The *error bias* represents the amount and direction of deviation relative to the target thus completing the information given by the *matching error*. These two measurements define the performance relying on error amplitude but do not carry any information about participants’ consistency across the 12 repetitions of the same target. In order to integrate such factor, the *variability* was evaluated. It has to be considered that this indicator does not depend from how close is the subject to the target but it rather evaluates how similarly participants match the same target. For the sake of clarity, if one subject matches a target always in the same position his/her *variability* will be slow, even if such position is highly incorrect. On the contrary, if the matched position is close to the ideal one but it changes from one trial to the other, the matching error will be slow, despite an increased *variability*.

### Statistics

Differences of *matching error*, *variability* and *error bias* between each DoF were determined using a one-way-ANOVA with p values < 0.05 being considered to be significant. In case of significance, a post-hoc Fisher LSD test was performed to confirm where the differences occurred among the three DoFs. A two-way ANOVA test (2 workspaces [LWS, SWS] x 3 DoFs [FE, AA, PS]) was then performed to investigate the effect of proprioceptive targets amplitude (LWS vs SMW) and DoFs.

## Results

### Anisotropy of proprioceptive acuity across the three degrees of freedom

**Fig 2:**
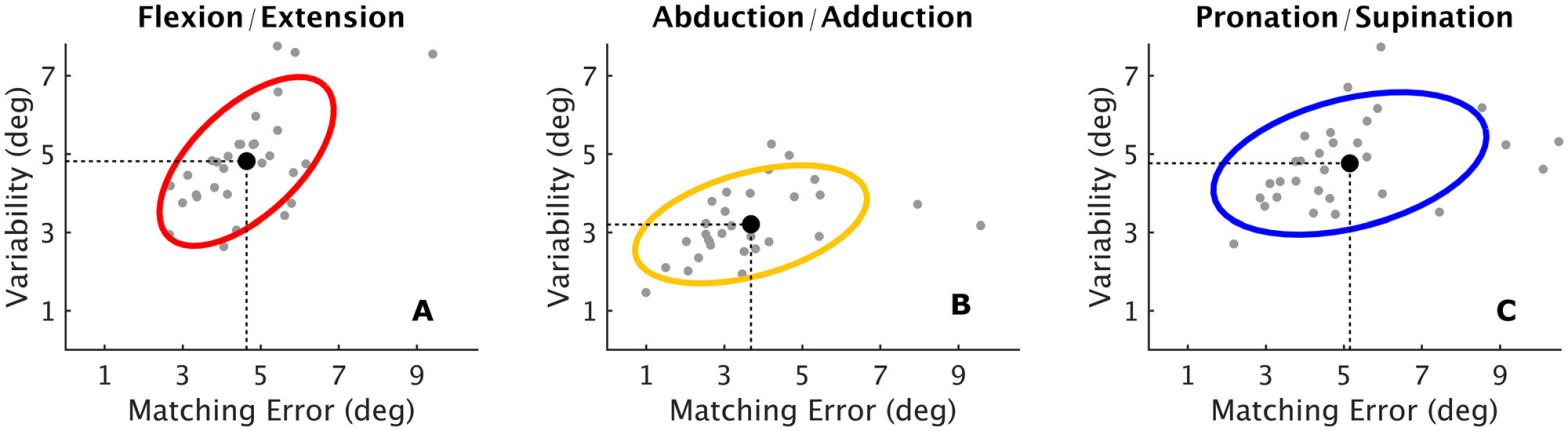
*Matching error* and *variability* for flexion/extension (A), abduction/adduction (B) and pronation/supination (C). The AA results the most accurate DoF (smallest *matching error*) and the most precise (lowest *variability*), compared with the other two (black dots). The three ellipses indicate the region that contains 75% of all samples (grey dots), obtained by averaging the 12 active trials for each subject (in total 30 grey dots represented in the figure for each Dof). The ellipses are obtained from the covariance matrix of the sampled data, where the eigenvectors of the covariance matrix represent the axes of the confidence ellipse, and thus models how the data was rotated. The eigenvalues on the other hand represent the variance of the data in the direction of these axes.

To understand, if proprioceptive acuity during active matching differed for the three tested DoF, we investigated the *matching error* and the *variability*. An Analysis of Variance procedure on matching error found a significant main effect for DoF (*F*_2,87_ = 5.31, *p* = 0.0067). A subsequent post-hoc analysis showed that Abduction/Adduction yielded the highest acuity (*ME* = 3.68 ± 0.32°), and was significantly different from FE (*ME* = 4.64 ± 0.24°, post hoc Fisher test, p = 0.040) and PS (*ME* = 5.15 ± 0.37°, Fisher, p = 0.0018), while no significant difference was found between FE and PS. A similar result was found for the *variability* (V) across the three DoFs (one-way ANOVA: *F*_2,87_ = 20.96, *p* < 0.001) where the AA showed the lowest *variability* (*V* = 3.05 ± 0.15°) when compared to FE (*V* = 4.59 ± 0.21°, Fisher, *p* < 0.001) and PS (*V* = 4.54 ± 0.18°, Fisher, *p* < 0.001). No significant differences were found between FE and PS. The respective data for *matching errors* and *variability* for each DoF and for all the subjects are shown in Fig 2: the figure depicts the confidence ellipses, each defining the region that contains 75% of all results, and provide an idea of how data are consistent and similarly distributed across the three tested degrees of freedom.

**Fig 3:**
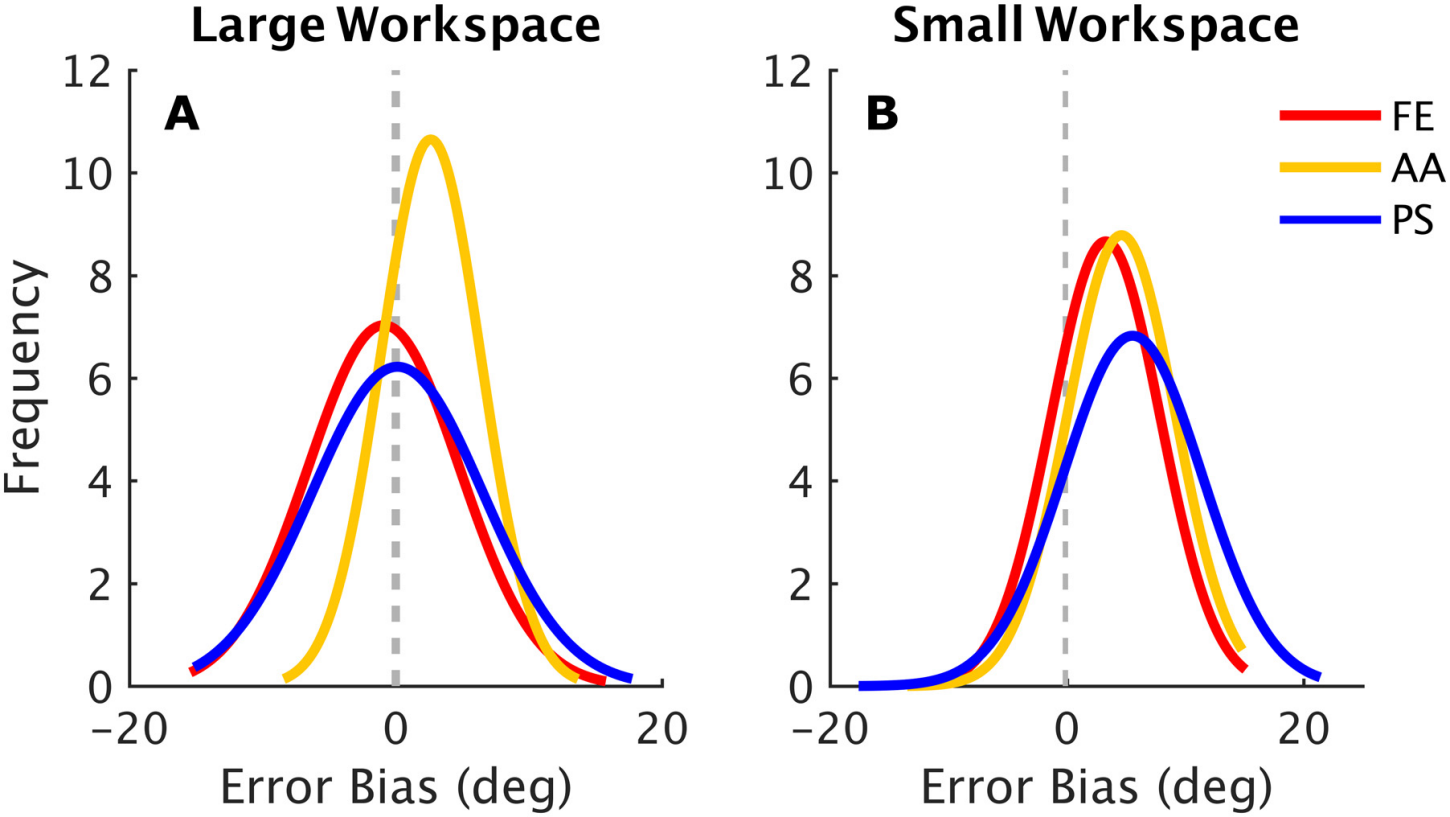
Probability density distributions for the *error bias* of all the three DoFs in the large (A) and the small (B) workspace. A distribution shifted to the left indicates subjects’ tendency of undershooting, vice versa, a distribution shifted to the right, representing predominance of positive *error biases*, indicates a predominant tendency of target overshooting.

To examine the tendency of Participants to overshoot and undershoot the probability density functions of *error bias* (EB) for each of the three DoFs was derived (Fig 3A and 3B). Without considering for now the difference between the two workspaces (large Vs small) which will be reported in the next results section, we can infer from an accurate observation of Fig 3 that in general the AA shows a tall narrow distribution shifted to the right (positive *error bias*), indicating a stronger predominance in target overshooting while FE and PS error bias distributions resulted to be more variable with no consistent predominance in over/undershooting of proprioceptive targets. The difference between *error biases* of the three DoFs were confirmed by a one-way ANOVA yielding a significant effect of DoF (*F*_2,87_ = 7.82, *p* = 0.00075). The results of the Fisher post-hoc revealed that the mean *error bias* for the AA (2.78 ± 0.39°) was significantly different from both FE (*EB* = −0.73 ± 0.61°; *p* < 0.001) and the PS (*EB* = −0.73 ± 0.61°; p = 0.00773) while, as for matching and variability, no significant differences were found between FE and PS.

### Mapping proprioception across the workspace

**Fig 4:**
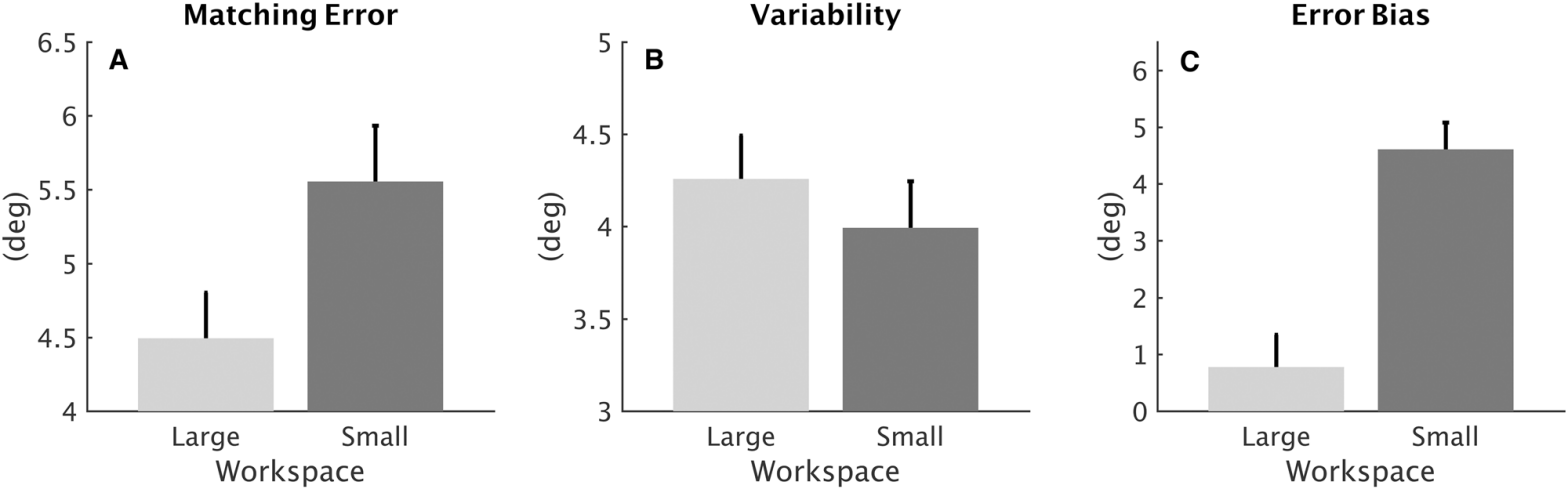
Overall difference, of the three DoFs, between performance in the large (LWS) and in the small (SWS) workspace for *matching error* (A), *variability* (B) *and error bias* (C).

**Fig 5:**
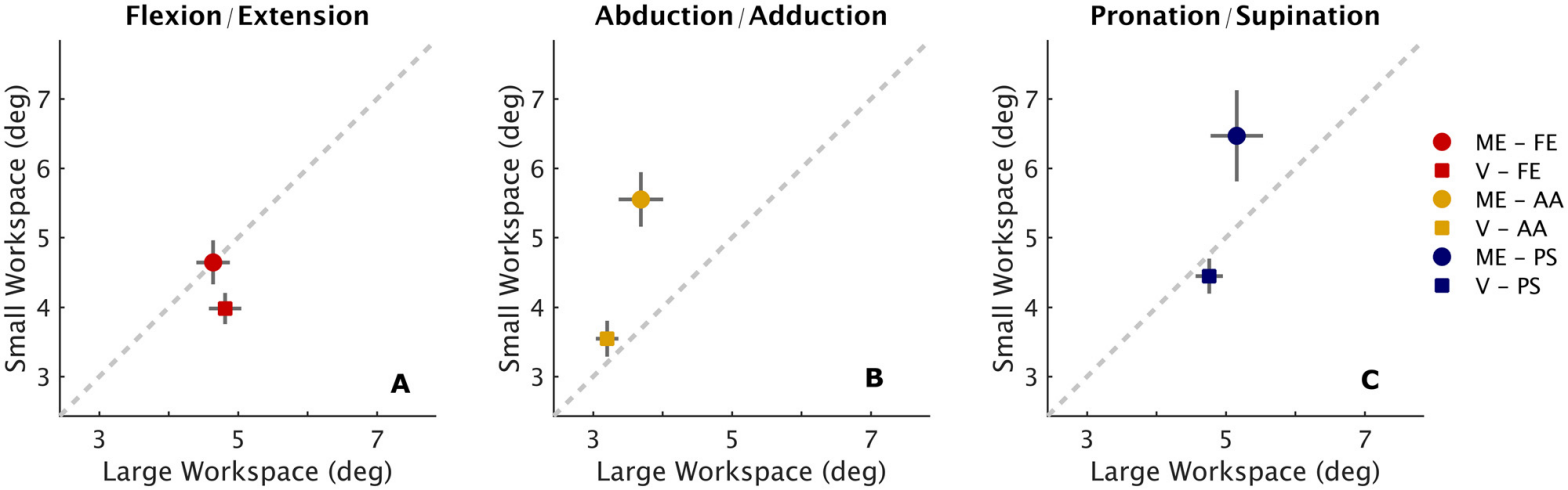
Differences between performance in large workspace (LSW) and small workspace (SWS) for flexion/extension (A), abduction/adduction (B) and pronation/supination. Errors which fall below the 45° line (equality line) indicate a worst performance in the LWS, whereas values which fall above the equality line indicate decrement in accuracy/precision in the SWS condition. Data points that fall directly on the equality line indicate that performance is equal in the two workspaces.

Figs 4 and 5 show the comparison between performance in the large (LWS) and in the small (SWS) workspace. Differences were found in *matching errors* which resulted to increase consistently from the LWS condition (*ME* = 4.49 ± 0.31°) to the SWS one (*ME* = 5.55 ± 0.38°) (Fig 4A). Statistical analysis revealed a main effect of the workspace (*F*_1,174_ = 9.8843, *p* = 0.000196) that is independent on the DoF (the interaction workspace x DoF did not reach significance). Fig 5 shows how the *matching error* is different in the two workspace, in particular for abduction/adduction deviation (Fig 5B) and pronation/supination (Fig 5C). Despite the significant difference in accuracy between small and large workspace, it was found that the *variability* remained almost constant for both the workspaces (Fig 4B) and for the three DoFs (Fig 5), highlighting that independently on the condition, subjects’ precision in matching movement did not deteriorate. The *error bias* changed significantly according to the amplitude of proprioceptive targets (*F*_1,174_ = 59.794, *p* < 0.001), as shown in Fig 4C, indeed, the probability density functions of the *error bias* in the small workspace (Fig 3B) are more shifted on the right side (positive *error biases*) thus yielding to target overshooting.

## Discussion

Clinicians have long recognised the necessity to use an accurate assessment tool for the diagnosis of proprioceptive dysfunction. Despite an extensive literature on proprioceptive assessment [11, 36, 37], an accepted standard protocol for the objective quantification of proprioceptive function is still missing. In the last few years haptic devices have been extensively used to implement novel methods to study proprioception but most of the contributions considered either proximal upper limb joints of such as the elbow [38] and shoulder [39, 40]), or lower limb joints such as the knee [41] and ankle [22]).

Our study focused on the distal arm that is essential for human fine motor control, manipulation and the haptic perception of objects. We proposed a robot-aided method that was able to provide reliable and quantitative data of wrist proprioceptive acuity.

Such method was based on the joint position matching test. Two types of position matching have commonly been used to assess proprioceptive acuity in clinical practice: the *ipsilateral* and the *contralateral* task. Participants enrolled in this study performed an *ipsilateral* matching task, in which the reference joint position had to be replicated using the same limb. This type of task is contrasted by the second commonly used task type, known as *contralateral* matching that involves matching of a reference joint angle with the opposite limb. Joint position matching using the opposite limb requires interhemispheric communication (or transfer) compared with *ipsilateral* matching that may represent a significant cognitive factor influencing performance in certain clinical populations [13]. However, *ipsilateral* matching requires to keep the previously experienced position in working memory before matching that position.

### Anisotropy of proprioceptive acuity

The main purpose of the present study was to systematically quantify the active wrist joint position sense across its three DoFs (FE, AA and PS). Here the term active implies that the matching gesture was performed actively by the person and not passively moved by the experimenter or a robot. We found that acuity for Abduction/Adduction is significantly higher in *both matching* error and *variability* than other DoFs (Flexion/Extension and Pronation/Supination). One reason for the observed anisotropy in proprioceptive acuity might be differences in receptors density [42, 43]; proprioceptive information comes from mechanoreceptors [44] comprising both Ruffini and Pacini types, as well as free nerve endings in ligaments, and the degree of innervation in the wrist ligaments was found to be highly variable across the different joints [45]. Immuno-histochemical studies of the wrist anatomy revealed a dense distribution of mechanoreceptors in the dorso-radial ligaments such as dorsal radiocarpal, dorsal intercarpal, and scapholunate interosseous, a medium density in the volar and volar-triquetral, while others such as the long radiolunate ligament are nearly void of mechanoreceptors [46, 47]. The highly innervated dorso-radial ligaments are involved during Abduction/Adduction, while the less innervated volar ligaments get primarily stressed during Flexion/Extension and Pronation/Supination. These differences in mechanoreceptor density and innervation might be responsible for the proprioceptive anisotropy found in our investigation. Our results on wrist proprioceptive anisotropy confirmed previous outcomes obtained by our group using the same robotic device [23] in which, instead of an ipsilateral joint position matching task to assess wrist joint position sense, they used a psychophysical method (unidirectional 2-alternative-forced-choice discrimination paradigm) to evaluate proprioceptive acuity using passive movements. Cappello et al. observed that Flexion/Extension and Abduction/Adduction are characterised by different proprioceptive thresholds (1.5° for AA and 2.2° for FE). This previous contribution on passive sensing is in line with what emerged from the present study on active sensing. However, the proprioceptive thresholds during active position sense testing are somewhat worse than during the active. A plausible explanation can be found in the substantial difference between the passive and active tasks: in the first experiment [23], subjects’ experienced only robot operated passive movements (stimuli) and they were asked to discriminate the largest one, while in our study, wrist proprioception was measured after an active voluntary wrist angular displacement towards a proprioceptive target, and while passive motion proprioception is based on afferent signals from peripheral receptors, active movements proprioception involves additional process of sensorimotor integration and motor control that actually increase the signal-to-noise ratio for proprioceptive perception present during motion [12].

### Position sense acuity varies across the wrist workspace

Previous studies have investigated if proprioceptive acuity presents variability across the workspace [28, 29, 48] and our results are consistent with these findings: the position error increases for smaller wrist angular displacements and accuracy becomes higher at the limits of joint range of motion, accordingly to what has been found for the shoulder [49, 50]. This is reasonable if we think that differences in proprioceptive functions may be related to joint geometry and to differences in the relative stretch of muscles and mechanoreceptors as the joint configuration changes: i.e. larger amplitude in joint angle ought to be better discriminated. The hypotheses is supported by a neurophysiological perspective that mechanoreceptors information is complemented by cutaneous afferents which are highly responsive for large movements [3, 51]. Furthermore Sanes et al. [52], reported that movement amplitude is directly proportional to the motor neuronal activation, suggesting that neuronal activation itself might be a further sensory feedback complementing proprioceptive information.

These results are also confirmed by the movement’s strategies observed in the two experimental conditions for the large and small workspaces. While the subjects did not show a predominant strategy for larger movement, target overshooting has been observed in the small the workspace (*error bias* always positive). Overshooting movements in small workspace can be attributed to the scarce amount of information from the mechanoreceptors which are not sufficiently active for small portions of the range of motion.

### Conclusion

In the present study we proposed an investigation on wrist joint proprioceptive acuity employing a robotic device to accurately quantify joint movements across a three dimensional workspace and by means of a reliable and repetitive protocol, easy to administer and which did limit the attention load for the subjects. The involved robotic device appeared to be a high suitable platform to assess wrist proprioceptive functions and it provided, clear, quantitative and precise information about the joint sensitivity, laying down the basis for a complete investigation of joint position sense. The findings herewith reported provide a systematic mapping of proprioceptive acuity across space and between the wrist DoFs, offering further insights on motor control strategies and performance during active position matching of proprioceptive targets that were still missing in rehabilitation practice. Results on thirty healthy participants showed that biomechanics and anatomical configuration of mechanoreceptors play a fundamental role in conveying information and allow the brain to represent the movements in absence of vision. The outcomes showed also that one of the three degrees of freedom is more accurate in position matching, furthermore it was found that the extension of the workspace is directly proportional to the level of accuracy in position matching, and information on proprioceptive target is highly correlated to the number of mechanoreceptors involved in the movement. The involved robotic device is a highly suitable platform to assess wrist proprioceptive function providing quantitative and precise information about the joint proprioceptive acuity. Moreover, the findings herewith reported provide a systematic mapping of proprioceptive acuity across space and between the wrist DoFs, offering insights on active position matching of proprioceptive targets that were still missing in current literature on proprioceptive assessment and which might find future applications in rehabilitation practice by correlating motor recovery and sensory deficits.
